# Characterization of the complete plastid genome of fireweed, *Epilobium angustifolium*, Shaanxi, China

**DOI:** 10.1080/23802359.2019.1688697

**Published:** 2019-11-13

**Authors:** Yu-Li Li, Shi-Yi Tang, Yu Dai, Yu-Ting Jiang, Bao-Guo Li

**Affiliations:** a College of Life Sciences, Shaanxi Key Laboratory for Animal Conservation, Northwest University, Xi’an, P. R. China;; b Center for Excellence in Animal Evolution and Genetics, Chinese Academy of Sciences, Kunming, P. R. China

**Keywords:** *Epilobium angustifolium*, chloroplast genome, phylogenetic tree

## Abstract

In this article, we studied the complete chloroplast genome of Fireweed, *Epilobium angustifolium*, an essential herbaceous perennial species of the genus *Epilobium* (Onagraceae), we used Illumina sequencing platform to characterize its whole plastid genome sequence. The results showed that its whole plastid genome is a typical qudaripartite circular molecule with 161,199 bp in total length, which contains a large single-copy region of 89,076 bp, a small single-copy region of 17,321 bp, and two inverted repeat regions of 27,401 bp. We identified 130 genes, 85 protein-coding genes, 37 tRNA, and 8 rRNA genes within this genome. The GC content in the chloroplast genome, LSC region, SSC region, and IR region were 38.1, 36.3, 33.1, and 42.7%, respectively. Phylogenetic analysis indicated that this plant was placed as a sister to the congeneric *Epilobium ulleungensis*, the two species were clustered into a clade with high bootstrap support.

*Epilobium angustifolium* (L.) Holub is a perennial herb that widely distributed worldwide (Husband and Schemske 1998). This species is often gathered and grown as medical or ornamental plants (Bertsch 1983). However, the numbers and ranges of its natural populations are often threatened by habitat fragmentation nowadays (Brook and Barnosky 2012). There is an urgent need to raise more attention to the conservation of this species. In plants, chloroplast DNA sequence genomes showed high copy numbers per cell and a much smaller size for complete sequencing, compared with nuclear genomes, as they can provide valuable phylogenetic signals on its taxonomic status (McNeal et al. 2006). In this study, we characterized the integrated chloroplast genome sequence of *Epilobium angustifolium* based on Illumina pair-end sequencing data; our results established a general foundation for future studies on population dynamics and conservation of this species.

We used the improved CTAB method to extract Genomic DNA from fresh leaves of an individual of *Epilobium angustifolium* sampled from Qinling Mountains (N 33.672494, E 107.997136; the specimen (s33091) was deposited at Northwest University) (Doyle and Doyle 1987). After that, the DNAs were subjected to Illumina sample preparation, and pair-read sequencing was indexed by the Illunmina Hiseq 2500 platform (Illumina, San Diego, CA, USA). We acquired 900,867 high quality reads in total, and the clean reads were assembled by the MIRA version 4.0.2 program[Q] (Chevreux et al. 2004) and MITObim version 1. 7 software[Q] (Hahn et al. 2013). The chloroplast genomes were annotated using the program DOGMA (Wyman et al. 2004), coupled with manual corrections for start and stop codons. Finally, the chloroplast genome sequences were deposited in GenBank (accession numbers, MN481508).

The complete chloroplast genome of *Epilobium angustifolium* was 161,199 bp in length, including a large single-copy (LSC) region of 89,076 bp and a small single copy (SSC) region of 17,321 bp. Both of them were separated by two inverted repeat regions (IRs) of 27,401 bp. The overall GC content of *Epilobium angustifolium* chloroplast genome was 38.1%, while the corresponding values of LSC, SSC, and IR regions were 36.3, 33.1, and 42.7%, respectively. The circular genome contained 130 genes, including 85 protein-coding genes, 37 tRNA, and 8 rRNA genes. A total 16 genes contained one intron including *atpF*, *ndhA*, *ndhB*, *nad2*, *rps16*, *rpoC1*, *rpl16*, *rpl2*, *petB*, *tRNI*, *tRNA-UGC*, *tRNL-GAU*, *tRNA-UAC*, *tRNL*, *tRNG*, and *tRNK*, and *clpP*, *ycf3* and *rps12* genes contained two intron.

Based on five species from *Nyssa* and *Oenothera*, three species from *Lagerstroemia*, each one species of the *Trapa*, *Sonneratia*, *Punica*, *Pemphis*, and *Epilobium*, we constructed the phylogenetic tree. All of 19 plastid sequences were aligned based on the software MAFFT with the default parameters (Kazutaka and Standley 2013). The phylogenetic analysis was conducted using the program RaxML with 1000 replicates (Figure 1). The results indicated the *Epilobium angustifolium* was the closest relative of the *Epilobium ulleungensis*, and the two species were clustered into a clade with high boostrap support. We believe that *Epilobium ulleungensis* chloroplast genome will provide valuable insight into utilization, conservation, and evolutionary histories for this species.

**Figure 1.**
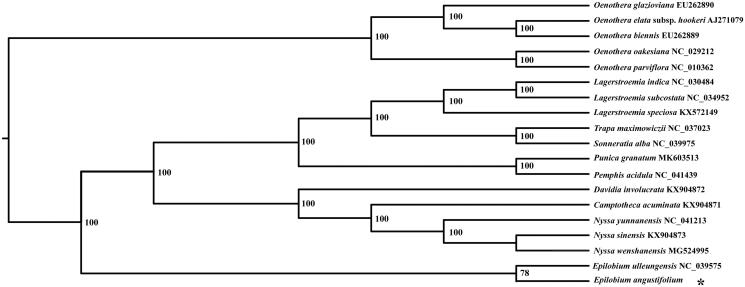
Phylogenetic tree based on 19 complete chloroplast genome sequences.
